# Functional Characterization of Dopamine Projections across Nucleus Accumbens Subregions in Reward-Related, Anxiety-Like, and Locomotor Behaviors

**DOI:** 10.1523/ENEURO.0002-26.2026

**Published:** 2026-07-15

**Authors:** Ximena I. Salinas-Hernández, Daphne Zafiri, Natasha N. Khan, Sevil Duvarci

**Affiliations:** Institute of Neurophysiology, Neuroscience Center, Goethe University, Frankfurt 60599, Germany

**Keywords:** anxiety, dopamine, movement, nucleus accumbens, reward

## Abstract

Mesolimbic dopamine (DA) neurons projecting to the nucleus accumbens (NAc) have traditionally been viewed as a functionally homogeneous reward pathway. However, recent evidence reveals functional heterogeneity, with DA projections to specific NAc subregions responding not only to rewards but also to aversive and safety-related stimuli. The extent to which DA projections across distinct NAc subregions differentially promote reward, aversion and anxiety-related behavior remains incompletely understood. To address this question, we performed optogenetic inhibition of DA terminals across different NAc subregions during real-time place preference and anxiety-related tests in predominantly male mice. We found that inhibition of DA terminals across all NAc subregions produced robust place avoidance, indicating that DA input to each subregion is similarly reinforcing and that its inhibition is aversive. In contrast, DA terminal inhibition resulted in subregion-specific effects on locomotor activity but had no acute effect on anxiety-like behavior. Together, these findings suggest that while DA projections to the NAc are uniformly reinforcing, they exhibit subregion-specific roles in regulating locomotor activity and have no acute effect in modulating anxiety, highlighting both common and diverging functions of distinct mesolimbic DA systems.

## Significance Statement

Mesolimbic dopamine system projecting to the nucleus accumbens (NAc) is associated with reward, motivation, aversion, and regulation of affective states. However, the functional contribution of projection-specific dopamine input to distinct NAc subregions is incompletely understood. Using optogenetic inhibition of dopamine terminals across NAc subregions, we show that dopamine input to each subregion uniformly promotes reward, as its suppression induces place avoidance. Conversely, dopamine terminal inhibition produces subregion-specific effects on movement and does not acutely affect anxiety-like behavior. These findings reveal that the mesolimbic dopamine projections share a common role in promoting reward but differ in their contributions to motor regulation. Our results provide important insights into functional architecture of the mesolimbic dopamine system, with important clinical implications for reward-related disorders.

## Introduction

Considerable evidence indicates that midbrain dopamine (DA) neurons are organized into distinct, largely nonoverlapping subpopulations defined by their projection targets, with each subpopulation mediating specific functions (Lynd-Balta and Haber, 1994; Lammel et al., 2008, 2011, 2012; Roeper, 2013; Beier et al., 2015; Lerner, et al., 2015; Menegas et al., 2015, 2017; Parker et al., 2016; Farassat et al., 2019). Among these, the mesolimbic DA system, consisting of DA neurons that project to the nucleus accumbens (NAc), plays a central role in reward processing and motivation (Wise, 2002; Berridge and Kringelbach, 2015; Schultz, 2016; Watabe-Uchida et al., 2017). DA neurons are known to encode reward prediction errors (RPEs), the difference between expected and actual rewards, that act as teaching signals for reinforcement learning (Schultz, 2016). Specifically, unexpected and better-than-expected rewards as well as reward-predicting cues lead to increased firing in DA neurons, while worse-than-expected and aversive outcomes inhibit DA neuron firing (Mirenowicz and Schultz, 1996; Schultz et al., 1997; Hollerman and Schultz, 1998; Schultz and Dickinson, 2000; Ungless et al., 2004; Bayer and Glimcher, 2005; Roesch et al., 2007; Cohen et al., 2012; Steinberg et al., 2013; Eshel et al., 2015, 2016; Schultz, 2016). DA terminals in NAc exhibit RPE signals and phasic activation in response to rewards (Parker et al., 2016; Menegas et al., 2017; de Jong et al., 2019; Yuan et al., 2019; Tsutsui-Kimura et al., 2020; Salinas-Hernández et al., 2023), consistent with the canonical role of the mesolimbic DA system in reward processing.

However, recent findings have begun to challenge the traditional view of the mesolimbic DA system as a functionally homogeneous circuit. Much evidence indicates that NAc is a heterogeneous structure composed of several subregions, each contributing differentially to motivated behaviors (Floresco, 2015; Castro and Bruchas, 2019). Furthermore, distinct subpopulations of DA neurons exhibit topographically organized projections to specific NAc subregions, suggesting a functional specialization within the mesolimbic pathway (Lammel et al., 2008, 2011, 2012; Roeper, 2013; Beier et al., 2015; Menegas et al., 2015; Farassat et al., 2019). In line with this, recent studies have demonstrated that DA projections to particular NAc subregions are activated not only by rewards but also by aversive and safety-related outcomes (de Jong et al., 2019; Yuan et al., 2019; Kutlu et al., 2023; Salinas-Hernández et al., 2023; Duvarci, 2024).

Despite emerging evidence of heterogeneity in the mesolimbic DA system, previous studies have consistently shown that excitation of VTA DA neurons, as well as their projections to NAc, are intrinsically reinforcing while their inhibition is aversive (Tsai et al., 2009; Tan et al., 2012; Danjo et al., 2014; McDevitt et al., 2014; Cai et al., 2020; Cardozo Pinto et al., 2025). Yet, the relative contribution of DA projections to distinct NAc subregions in promoting reward versus aversion remains incompletely understood. Furthermore, VTA DA neurons and their projections to NAc have been implicated in affective states such as stress and depression (Heshmati and Russo, 2015; Willmore et al., 2022; Nestler and Russo, 2024). However, whether DA projections to NAc modulate anxiety-like behavior, particularly in a subregion-specific manner, is largely unknown.

In this study, we characterize the differential functional contributions of DA projections to distinct subregions of the NAc in regulating reward-related, anxiety-like, and locomotor behaviors. To this end, we performed optogenetic inhibition of DA terminals within defined NAc subregions to causally examine their functional roles in predominantly male mice. First, we evaluated whether these DA projections are intrinsically reinforcing or aversive using a real-time place preference paradigm. Next, we tested whether these DA projections regulate locomotor behavior in an open field test, to evaluate their role in spontaneous movement. Finally, we examined whether these DA projections modulate anxiety-like behaviors using two well-established anxiety tests.

## Materials and Methods

### Subjects

All procedures were conducted in accordance with the guidelines of the German Animal Protection Act and were approved by the local authorities (Regierungspräsidium Darmstadt). Adult male (*n* = 43) and female (*n* = 6) heterozygous DAT-Cre mice (Zhuang et al., 2005; backcrossed with C57BL/6N) aged older than 3 months at the start of experiments were used. Littermate mice were assigned to experimental versus control groups. All mice were individually housed on a 12 h light/dark cycle. All experiments were performed during the light cycle.

### Viral constructs

AAV5-EF1a-DIO-eArch3.0-EYFP and AAV5-EF1a-DIO-EYFP (Mattis et al., 2011; gifts from Karl Deisseroth) were obtained from the University of North Carolina Vector Core.

### Surgical procedures

Animals were anesthetized using isoflurane (1–2%) and placed in a stereotaxic frame. At the onset of anesthesia, all animals received intraperitoneal injections of atropine (0.05 mg/kg) and subcutaneous injections of carprofen (4 mg/kg) and dexamethasone (2 mg/kg). Eye gel was applied on the eyes to prevent dehydration of the cornea. Lidocaine cream was applied on the scalp as local anesthetic. The animal's temperature was maintained for the duration of the surgical procedure using a heating blanket. Anesthesia levels were monitored throughout the surgery and the concentration of isoflurane adjusted so that the breathing rate never fell below 1 Hz.

Mice were injected bilaterally with 0.5–1 µl of AAV5-EF1a-DIO-eArch3.0-EYFP (final titer 5.6 × 10^12^ pp per ml) or AAV5-EF1a-DIO-EYFP (final titer 4 × 10^12^ pp per ml) per hemisphere in the VTA (3.2 mm posterior to bregma, 0.5 mm lateral to the midline and 4.5 mm ventral to bregma) at 50 nl/min using a 10 µl syringe with a 33-gauge needle controlled by an injection pump. The needle was left in place for an additional 10–15 min before slowly being withdrawn. Following infusion of the virus, optical fibers (200 µm core diameter, 0.22 NA, Thorlabs) were implanted bilaterally above the NAc (amNAc: 1.7 mm anterior and 0.8 mm lateral to bregma; pmNAc: 1.0 mm anterior and 0.8 mm lateral to bregma; lNAc: 1.1 mm anterior and 1.8 mm lateral to bregma and to a depth of 3.9–4.1 mm below bregma; vNAc: 1.3 mm anterior, 1.1 mm lateral to bregma and to a depth of 4.75 mm below bregma). The optical fibers were then anchored to the skull using skull screws and dental cement (Paladur).

### Behavior

#### Real-time place preference test

Real-time place preference test was conducted in a custom-made chamber (50 × 50 × 50 cm, wooden gray box) divided into two compartments. The test consisted of two 10 min phases (Fig. 2*A*). During the first phase, one side of the chamber was randomly assigned as the laser ON side. Mice were individually connected to the patch cords and placed in the laser OFF side of the chamber. Each time the mouse entered the laser ON side, laser light was delivered until the mouse crossed back to the OFF side. In the second phase, the sides were switched and the previously laser OFF side became laser ON side in order to counterbalance each side. As the avoidance of the laser ON side was comparable between the two phases in all four groups (amNAc, *p* = 0.27; pmNAc, *p* = 0.53; lNAc, *p* = 0.74; vNAc, *p* = 0.055), the data from the two phases were averaged for further analysis.

#### Open field test

A custom-made chamber (50 × 50 × 50 cm, wooden gray box) was used for the open field test. For testing anxiety-like behavior, the chamber was divided into a central area (center 25 × 25 cm) and an outer area (periphery; Fig. 5*A*). The open field test consisted of a 9 min session with three alternating 3 min epochs (OFF-ON-OFF epochs) in which laser was delivered during the ON epoch (Figs. 5*A*, 6*A*).

#### Elevated plus maze

Elevated plus maze (EPM) consisted of two open arms (30 × 5 cm), two closed arms (30 × 5 × 15 cm), and a central area (5 × 5 × 5 cm). The maze was placed 40 cm above the floor. Mice were individually connected to the patch cords and placed in the center. The test consisted of a 9 min session with three alternating 3 min epochs (OFF-ON-OFF epochs) in which laser was delivered during the ON epoch (Fig. 8*A*).

### Optogenetic experiments

For bilateral optogenetic inhibition during behavior, the implanted optical fibers (200 µm core diameter, 0.22 NA, Thorlabs) were connected to 200 µm patch cords (Thorlabs) with zirconia sleeves, and the patch cords were connected to a light splitting rotary joint (FRJ 1×2i, Doric Lenses) that was connected to a laser with a 200 µm patch cord (Thorlabs). Yellow light was delivered from a DPSS 594 nm laser (Omicron). Laser power at the tip of the optic fiber was 5–10 mW. During the real-time place preference/avoidance test, each time the mouse entered the laser ON side laser light was delivered until the mouse crossed back to the OFF side. The open field and EPM tests consisted of three alternating 3 min epochs (OFF-ON-OFF epochs) in which laser was delivered during the ON epoch.

### Histology

At the end of the experiments, mice were deeply anesthetized with sodium pentobarbital and were transcardially perfused with 4% paraformaldehyde and 15% picric acid in phosphate-buffered saline (PBS). Brains were removed and post-fixed overnight and coronal brain slices (60–100 µm) were sectioned using a vibratome (VT1000S, Leica). Standard immunohistochemical procedures were performed on free-floating brain slices. Briefly, sections were rinsed with PBS and then incubated in a blocking solution (10% horse serum, 0.5% Triton X-100, and 0.2% BSA in PBS) for 1 h at room temperature. Slices were then incubated in a carrier solution (1% horse serum, 0.5% Triton X-100, and 0.2% BSA in PBS) containing the primary antibody overnight at room temperature. The next day, the sections were washed in PBS and then incubated in the same carrier solution containing the secondary antibody overnight at room temperature. The following primary antibodies were used: polyclonal rabbit anti-tyrosine hydroxylase (TH, catalog #657012, 1:1,000, Calbiochem), monoclonal mouse anti-TH (catalog #MAB318, 1:1,000, Millipore), polyclonal guinea pig anti-TH (catalog #213004, 1:1,000, Synaptic Systems), polyclonal rabbit anti-GFP (catalog #A11122, 1:1,000, Life Technologies), polyclonal chicken anti-GFP (catalog #AB13970, Abcam). The following secondary antibodies were used: Alexa Fluor 568 goat anti-rabbit (catalog #A11011, 1:1,000, Thermo Fisher Scientific, Invitrogen), Alexa Fluor 568 goat anti-mouse (catalog #A11004, 1:1,000, Thermo Fisher Scientific, Invitrogen), Alexa Fluor 568 goat anti-guinea pig (catalog #A11075, 1:1,000, Invitrogen), Alexa Fluor 488 goat anti-rabbit (catalog #A11008, 1:1,000, Thermo Fisher Scientific, Invitrogen), Alexa Fluor 488 goat anti-chicken (catalog #AB150173, 1:1,000, Abcam). For DAPI staining, sections were incubated for 10 min in 0.1 M PBS containing 0.02% DAPI (catalog #D1306, Molecular Probes, Invitrogen). Finally, all sections were washed with PBS, mounted on slides embedded with a mounting medium for fluorescence (VECTASHIELD, Vector Laboratories) and coverslipped.

### Statistics

Data were statistically analyzed using GraphPad Prism (GraphPad Software). All statistical tests were two-tailed and had an *α* level of 0.05. All error bars show SEM. All ANOVAs were followed by Bonferroni post hoc tests if significant main or interaction effects were detected. No statistical methods were used to predetermine sample size, but our sample sizes were similar to those generally used in the field. Animals for the experimental and control groups were matched from littermate mice. All results were obtained using groups of mice that were run in several cohorts.

## Results

### Inhibition of DA neuron terminals induces place avoidance in all NAc subregions

**Figure 1.**
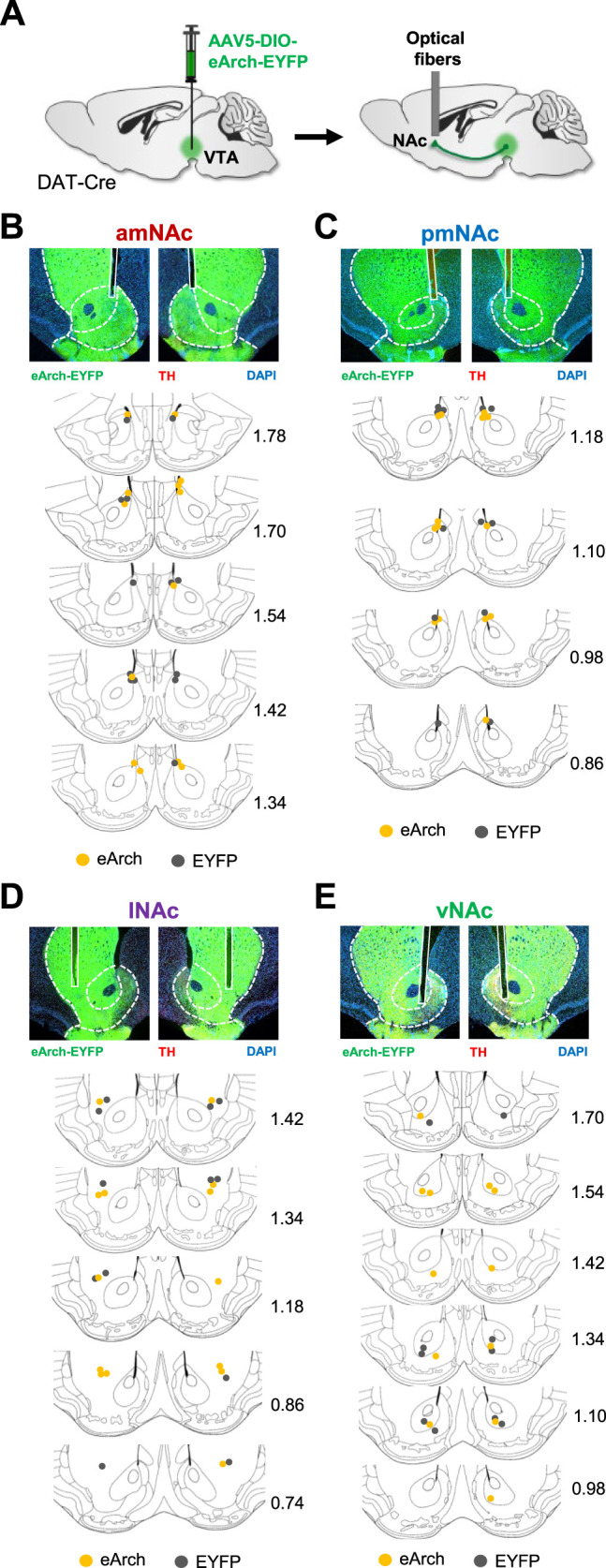
Histological verification of optical fiber placements in NAc subregions. ***A***, Schematic of the surgical procedure showing virus injection in the VTA and optical fiber implantation in the NAc. ***B–E***, Top, Example histological images showing optical fiber placements and Cre-dependent expression of eArch-EYFP at DA terminals (green) along with immunostaining for TH (red) and DAPI (blue) staining in the amNAc (***B***), pmNAc (***C***), lNAc (***D***), and vNAc (***E***). White vertical tracks indicate the bilateral optical fiber placements in the NAc. Bottom, Schematic coronal sections showing the bilateral placement of the tips of the optical fibers for eArch (yellow) and EYFP (gray) mice in amNAc (***B***), pmNAc (***C***), lNAc (***D***), and vNAc (***E***) groups. Numbers represent distance anterior to the bregma.

While DA neurons projecting to different subregions of the NAc have been shown to differentially encode threats and safety signals, their responses to rewards have been largely uniform across these subregions (de Jong et al., 2019; Yuan et al., 2019; Kutlu et al., 2023; Salinas-Hernández et al., 2023; Duvarci, 2024). We therefore sought to investigate whether DA projections to distinct NAc subregions differentially influence place preference/avoidance behavior. To address this question, we optogenetically inhibited DA neuron terminals in NAc subregions during a real-time place preference test. To this end, we injected a Cre-dependent adeno-associated virus (AAV) expressing either the light-activated inhibitory opsin archaerhodopsin fused with enhanced yellow fluorescent protein (eArch-EYFP) or EYFP only (EYFP control) into the VTA of transgenic mice expressing Cre recombinase under the control of the dopamine transporter (Dat) promoter (DAT-Cre mice; Fig. 1*A*). In these mice, the expression of Cre is highly selective for DA neurons (Lammel et al., 2015). Optical fibers were implanted bilaterally above the NAc (Fig. 1) to allow for selective inhibition of VTA DA terminals. eArch has previously been shown to effectively suppress DA terminal activity in the NAc in vivo (Luo et al., 2018). Notably, recent studies have identified DA projections to four distinct NAc subregions—anteromedial, posteromedial, lateral, and ventral—as differentially encoding threats and safety cues (de Jong et al., 2019; Yuan et al., 2019; Salinas-Hernández et al., 2023). To examine the distinct contribution of these DA projections, the optical fibers were implanted above the anteromedial (amNAc), posteromedial (pmNAc), lateral (lNAc), and ventral (vNAc) NAc subregions (Fig. 1*B*–*E*).

**Figure 2.**
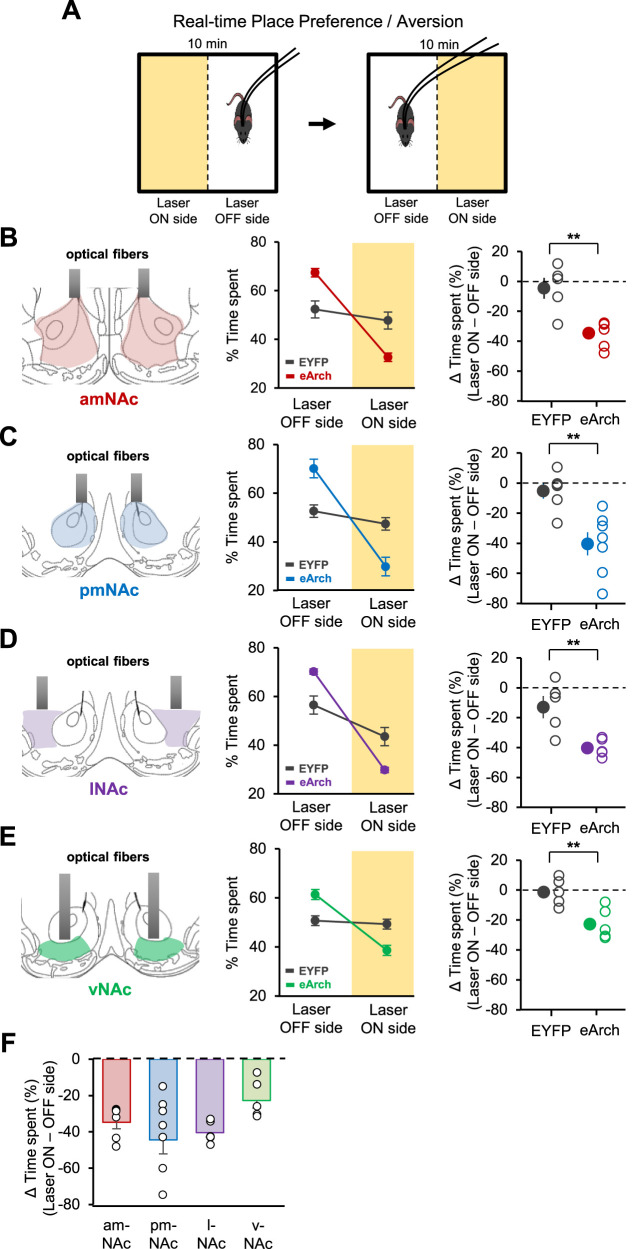
Inhibition of DA neuron terminals causes real-time place avoidance in all subregions of NAc. ***A***, Schematic of the real-time place preference/avoidance test. ***B***, Left, Schematic of optogenetic inhibition in amNAc. Middle, Percent time eArch- (*n* = 6) and EYFP-expressing (*n* = 5) mice spent in laser OFF and laser ON sides. Right, Difference between the percent of time mice spent in laser ON minus laser OFF side. eArch-expressing mice spent significantly less time in the laser ON side compared with EYFP mice (unpaired *t* test, *t*_(9)_ = 4.06, **p = 0.002), indicating inhibition of DA terminals in amNAc resulted in place avoidance. ***C***, Left, Schematic of optogenetic inhibition in pmNAc. Middle, Percent time eArch- (*n* = 7) and EYFP-expressing (*n* = 6) mice spent in laser OFF and laser ON sides. Right, Difference between the percent of time mice spent in laser ON minus laser OFF side. eArch-expressing mice spent significantly less time in the laser ON side compared with EYFP mice (unpaired *t* test, *t*_(11)_ = 3.66, **p = 0.003), indicating inhibition of DA terminals in pmNAc resulted in place avoidance. ***D***, Left, Schematic of optogenetic inhibition in lNAc. Middle, Percent time eArch- (*n* = 6) and EYFP-expressing (*n* = 5) mice spent in laser OFF and laser ON side. Right, Difference between the percent of time mice spent in laser ON minus laser OFF side. eArch-expressing mice spent significantly less time in the laser ON side compared with EYFP mice (unpaired *t* test, *t*_(9)_ = 3.83, **p = 0.004), indicating inhibition of DA terminals in lNAc resulted in place avoidance. ***E***, Left, Schematic of optogenetic inhibition in vNAc. Middle, Percent time eArch- (*n* = 6) and EYFP-expressing (*n* = 5) mice spent in laser OFF and laser ON sides. Right, Difference between the percent of time mice spent in laser ON minus laser OFF side. eArch-expressing mice spent significantly less time in the laser ON side compared with EYFP mice (unpaired *t* test, *t*_(9)_ = 3.67, **p = 0.005), indicating inhibition of DA terminals in vNAc resulted in place avoidance. ***F***, Comparison of the difference between the percent time eArch-expressing mice spent in laser ON minus laser OFF side between the four groups. The four groups exhibited comparable level of place avoidance (one-way ANOVA, *F*_(3,24)_ = 2.55, *p* = 0.08). Error bars represent mean ± SEM.

To assess place preference or avoidance behavior, mice were placed in a chamber with two compartments, with one side assigned as “laser ON” compartment and the other side as “laser OFF.” Each time the mouse entered the laser ON side, laser light was delivered to inhibit DA terminals in NAc until the mouse crossed back to the OFF side (Fig. 2*A*). In all four groups, mice expressing eArch (amNAc: *n* = 6, 5 males and 1 female; pmNAc: *n* = 7, 6 males and 1 female; lNAc: *n* = 6, 5 males and 1 female; vNAc: *n* = 6, 4 males and 2 females) spent significantly less time in the laser ON side compared with the OFF side, whereas EYFP-expressing control mice (amNAc: *n* = 5 males; pmNAc: *n* = 6 males; lNAc: *n* = 5 males; vNAc: *n* = 5, 4 males and 1 female) showed no such preference and spent similar time in both compartments (Fig. 2*B*–*E*). Two-way repeated-measures ANOVA revealed significant group × trial interactions for each subregion tested: amNAc (*F*_(1,9)_ = 16.55, *p* = 0.0028), pmNAc (*F*_(1,11)_ = 13.45, *p* = 0.0037), lNAc (*F*_(1,9)_ = 14.71, *p* = 0.0040), and vNAc (*F*_(1,9)_ = 31.57, *p* = 0.0051). These results indicate that inhibition of DA terminals across all NAc subregions induces place avoidance and thus, aversion. Importantly, the extent of place avoidance in eArch-expressing mice was comparable between the four groups (Fig. 2*F*), suggesting that inhibition of DA terminals in each NAc subregion is equally aversive.

**Figure 3.**
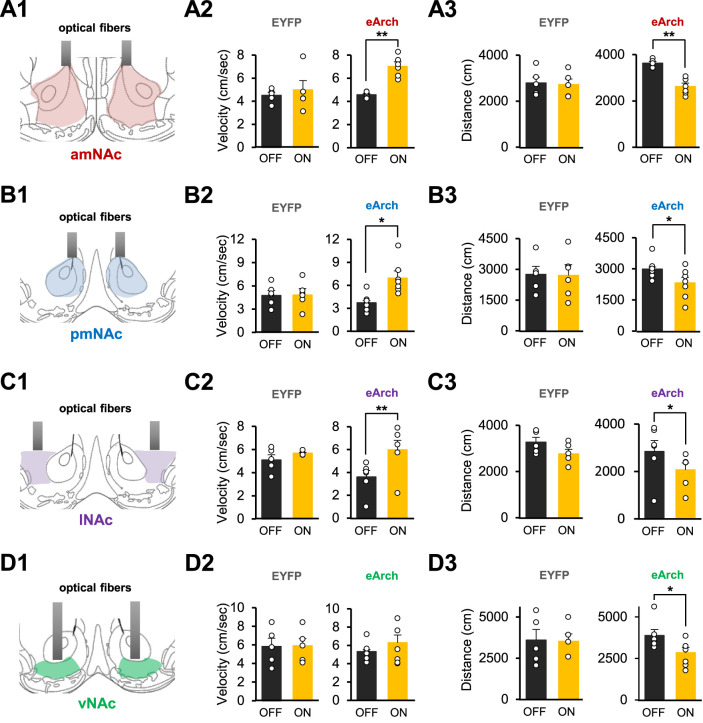
Effect of DA terminal inhibition in NAc subregions on velocity and distance traveled during the real-time place avoidance. ***A1***, Schematic of optogenetic inhibition in amNAc. ***A2***, Velocity in laser OFF and ON sides for EYFP- (*n* = 5; paired *t* test, *t*_(4)_ = 0.65, *p* = 0.55) and eArch-expressing mice (*n* = 6; paired *t* test, *t*_(5)_ = 6.83, **p = 0.001). ***A3***, Distance traveled in laser OFF and ON sides for EYFP- (paired *t* test, *t*_(4)_ = 2.37, *p* = 0.076) and eArch-expressing mice (paired *t* test, *t*_(5)_ = 5.70, **p = 0.0023). ***B1***, Schematic of optogenetic inhibition in pmNAc. ***B2***, Velocity in laser OFF and ON sides for EYFP- (*n* = 6; paired *t* test, *t*_(5)_ = 0.038, *p* = 0.97) and eArch-expressing mice (*n* = 7; paired *t* test, *t*_(6)_ = 3.06, *p = 0.02). ***B3***, Distance traveled in laser OFF and ON sides for EYFP- (paired *t* test, *t*_(5)_ = 0.34, *p* = 0.74) and eArch-expressing mice (paired *t* test, *t*_(6)_ = 3.61, *p = 0.011). ***C1***, Schematic of optogenetic inhibition in lNAc. ***C2***, Velocity in laser OFF and ON sides for EYFP- (*n* = 5; paired *t* test, *t*_(4)_ = 1.25, *p* = 0.27) and eArch-expressing mice (*n* = 6; paired *t* test, *t*_(5)_ = 6.25, **p = 0.0015). ***C3***, Distance traveled in laser OFF and ON sides for EYFP- (paired *t* test, *t*_(4)_ = 1.99, *p* = 0.11) and eArch-expressing mice (paired *t* test, *t*_(5)_ = 3.16, *p = 0.025). ***D1***, Schematic of optogenetic inhibition in vNAc. ***D2***, Velocity in laser OFF and ON sides for EYFP- (*n* = 5; paired *t* test, *t*_(4)_ = 0.29, *p* = 0.78) and eArch-expressing mice (*n* = 6; paired *t* test, *t*_(5)_ = 1.34, *p* = 0.23). ***D3***, Distance traveled in laser OFF and ON sides for EYFP- (paired *t* test, *t*_(5)_ = 0.30, *p* = 0.77) and eArch-expressing mice (paired *t* test, *t*_(5)_ = 3.25, *p = 0.022). Error bars represent mean ± SEM.

**Figure 4.**
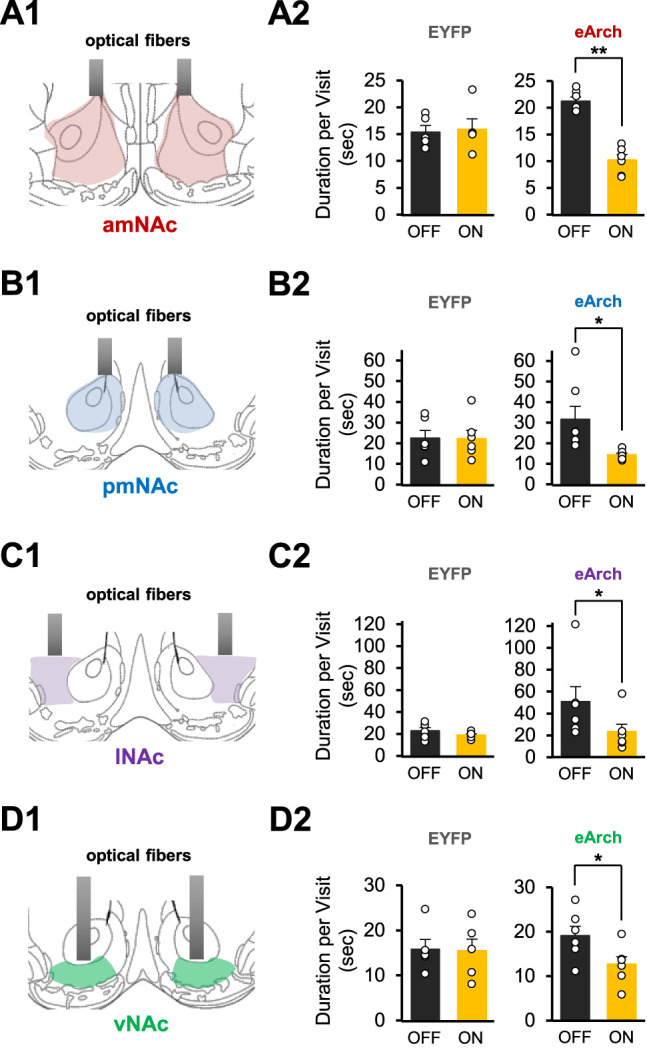
Effect of DA terminal inhibition in NAc subregions on the duration animals spent per visit to the OFF and ON sides during the real-time place avoidance. ***A1***, Schematic of optogenetic inhibition in amNAc. ***A2***, Duration per visit in laser OFF and ON sides for EYFP- (*n* = 5; paired *t* test, *t*_(4)_ = 0.36, *p* = 0.73) and eArch-expressing mice (*n* = 6; paired *t* test, *t*_(5)_ = 6.75, **p = 0.0011). ***B1***, Schematic of optogenetic inhibition in pmNAc. ***B2***, Duration per visit in laser OFF and ON sides for EYFP- (*n* = 6; paired *t* test, *t*_(5)_ = 0.12, *p* = 0.90) and eArch-expressing mice (*n* = 7; paired *t* test, *t*_(6)_ = 2.58, *p = 0.04). ***C1***, Schematic of optogenetic inhibition in lNAc. ***C2***, Duration per visit in laser OFF and ON sides for EYFP- (*n* = 5; paired *t* test, *t*_(4)_ = 1.39, *p* = 0.23) and eArch-expressing mice (*n* = 6; paired *t* test, *t*_(5)_ = 3.36, *p = 0.02). ***D1***, Schematic of optogenetic inhibition in vNAc. ***D2***, Duration per visit in laser OFF and ON sides for EYFP- (*n* = 5; paired *t* test, *t*_(4)_ = 0.25, *p* = 0.81) and eArch-expressing mice (*n* = 6; paired *t* test, *t*_(5)_ = 2.87, *p = 0.034). Error bars represent mean ± SEM.

Interestingly, further behavioral analysis revealed that inhibition of DA projections across NAc subregions affected movement during the place preference test. Specifically, mice expressing eArch showed increased velocity in the laser ON side in amNAc, pmNAc, and lNAc, but not in vNAc, groups (Fig. 3*A2*–*D2*). It is possible that this increase in locomotor activity may contribute to the observed place avoidance as increased nonspecific movement during laser ON periods could push the mice away from the ON side. However, this interpretation is not supported by the results from the vNAc group, which exhibited place avoidance comparable to that of the other groups (Fig. 2*F*) without any accompanying increase in velocity (Fig. 3*D2*). To further address this, we quantified the total distance traveled in each side and found that eArch-expressing mice in all four groups traveled significantly less distance in the ON side compared with the OFF side (Fig. 3*A3*–*D3*), indicating that all four groups indeed avoided the laser ON side. Finally, we analyzed the average duration of individual visits to the ON and OFF sides and found that eArch-expressing mice in all four groups spent significantly less time per visit to the ON side compared with the OFF side (Fig. 4*A2*–*D2*), providing additional evidence for place avoidance. Together, these results indicate that although optogenetic inhibition increased velocity in some groups, this effect did not account for the overall avoidance behavior.

### The effect of DA terminal inhibition on movement differs across NAc subregions

**Figure 5.**
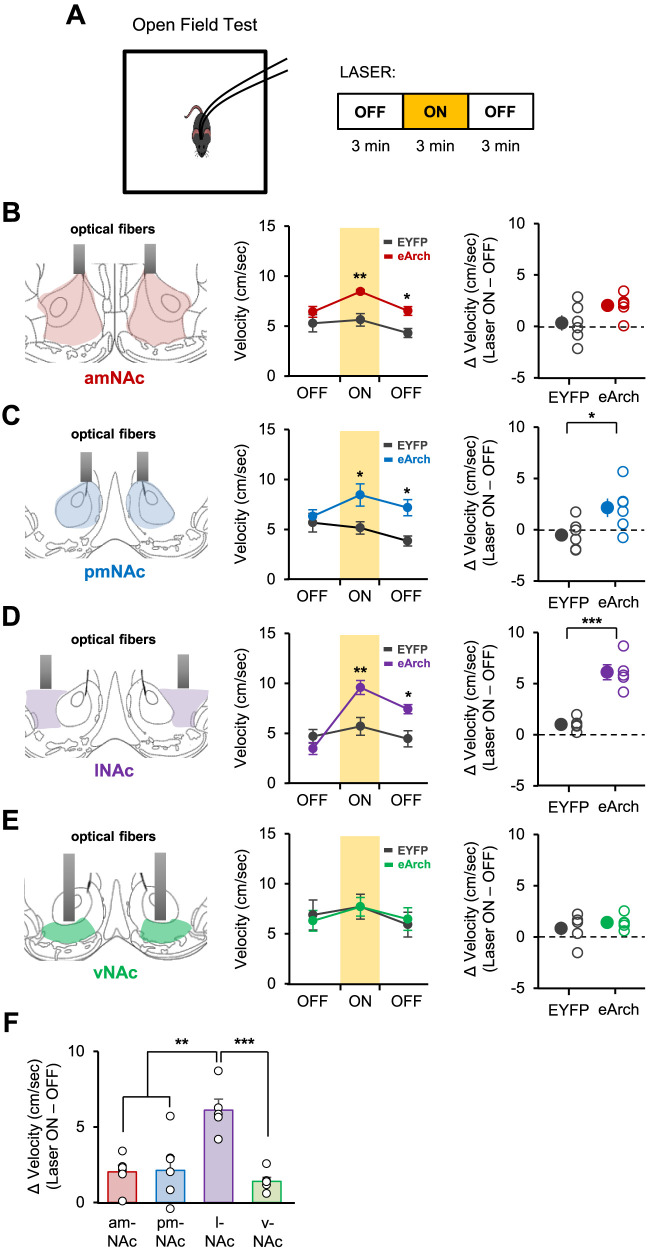
The effect of DA terminal inhibition on movement differs between NAc subregions. ***A***, Schematic of the open field test and optogenetic inhibition protocol. ***B***, Left, Schematic of optogenetic inhibition in amNAc. Middle, Velocity of eArch- (*n* = 6) and EYFP-expressing (*n* = 6) mice during laser OFF and laser ON epochs of the test. Right, Difference between the velocity of mice in laser ON minus laser OFF epochs. The two groups showed comparable velocity (unpaired *t* test, *t*_(10)_ = 1.96, *p* = 0.07). ***C***, Left, Schematic of optogenetic inhibition in pmNAc. Middle, Velocity of eArch- (*n* = 6) and EYFP-expressing (*n* = 6) mice during laser OFF and laser ON epochs of the test. Right, Difference between the velocity of mice in laser ON minus laser OFF epochs. eArch-expressing mice exhibited increased velocity during the ON epoch compared with the EYFP mice (unpaired *t* test, *t*_(10)_ = 2.47, *p = 0.03). ***D***, Left, Schematic of optogenetic inhibition in lNAc. Middle, Velocity of eArch- (*n* = 5) and EYFP-expressing (*n* = 5) mice during laser OFF and laser ON epochs of the test. Right, Difference between the velocity of mice in laser ON minus laser OFF epochs. eArch-expressing mice exhibited increased velocity during the ON epoch compared with the EYFP mice (unpaired *t* test, *t*_(8)_ = 6.52, ***p = 0.0002). ***E***, Left, Schematic of optogenetic inhibition in vNAc. Middle, Velocity of eArch- (*n* = 6) and EYFP-expressing (*n* = 5) mice during laser OFF and laser ON epochs of the test. Right, Difference between the velocity of mice in laser ON minus laser OFF epochs. The two groups showed comparable velocity (unpaired *t* test, *t*_(9)_ = 0.85, *p* = 0.41). ***F***, Comparison of the difference between the velocity of eArch-expressing mice in laser ON minus laser OFF side between the four groups. lNAc group showed a higher increase in velocity compared with the other three groups (one-way ANOVA, significant effect of group: *F*_(3,22)_ = 10.69, *p* = 0.0002). Error bars represent mean ± SEM.

DA neuron activity is known to regulate locomotor behavior (Schultz, 2007). Given that we observed changes in movement during the place preference test, we next sought to test this effect more directly. Specifically, we examined whether optogenetic inhibition of DA terminals within NAc alters locomotor activity in an open field test and whether these effects differ across NAc subregions. To this end, we optogenetically inhibited DA terminals in NAc, while mice explored an open field arena (Fig. 5*A*). To examine the differential effects across NAc subregions, optogenetic inhibition was performed in amNAc, pmNAc, lNAc, and vNAc (Fig. 1*B*–*E*) as described above.

Open field test consisted of a session with three alternating epochs (OFF-ON-OFF epochs), during which laser was delivered in the ON epoch (Fig. 5*A*). Mice expressing eArch exhibited increased velocity during the laser ON epoch in the amNAc (*n* = 6 males), pmNAc (*n* = 6 males), and lNAc (*n* = 5 males), but not in vNAc (*n* = 6, 4 males and 2 females), groups (Fig. 5*B*–*E*), consistent with the locomotor effects observed during the place preference test. In contrast, control mice expressing EYFP (amNAc: *n* = 6 males; pmNAc: *n* = 6 males; lNAc: *n* = 5 males; vNAc: *n* = 5, 4 males and 1 female) showed no significant change in movement across epochs (Fig. 5*B*–*E*). Two-way repeated-measures ANOVA revealed significant effect of group or group × trial interaction for amNAc (*F*_(1,20)_ = 11.36, *p* = 0.0071), pmNAc (*F*_(1,20)_ = 6.00, *p* = 0.03), and lNAc (*F*_(2,16)_ = 32.74, *p* < 0.0001), but not for vNAc (*F*_(1,18)_ = 0.00006, *p* = 0.99). Interestingly, eArch-expressing mice in the amNAc, pmNAc, and lNAc groups continued to show elevated velocity during the second OFF epoch (Fig. 5*B*–*E*), suggesting that DA terminal inhibition in the NAc had a prolonged influence on movement.

The differential effects of the optogenetic inhibition across NAc subregions were further supported by comparing the velocity change between the first OFF and ON epochs in eArch- versus EYFP-expressing mice (Fig. 5*B*–*E*). Notably, DA terminal inhibition led to a significantly larger increase in velocity in lNAc compared with the amNAc, pmNAc, and vNAc, which did not differ significantly from each other (Fig. 5*F*). Together, these results indicate that DA projections to NAc influence movement and further reveal the subregion-specific effects of DA input, with the strongest effect in lNAc.

### Inhibition of DA terminals across NAc does not acutely affect anxiety-like behavior in the open field

Previous studies have demonstrated that DA projections to distinct NAc subregions differentially encode aversive stimuli and threats (de Jong et al., 2019; Yuan et al., 2019; Salinas-Hernández et al., 2023). It remains to be determined whether these projections that encode threats also modulate anxiety and whether inhibition of these terminals has an effect on anxiety-like behaviors. To this end, we optogenetically inhibited DA terminals across different NAc subregions (Fig. 1*B*–*E*), while mice explored an open field.

**Figure 6.**
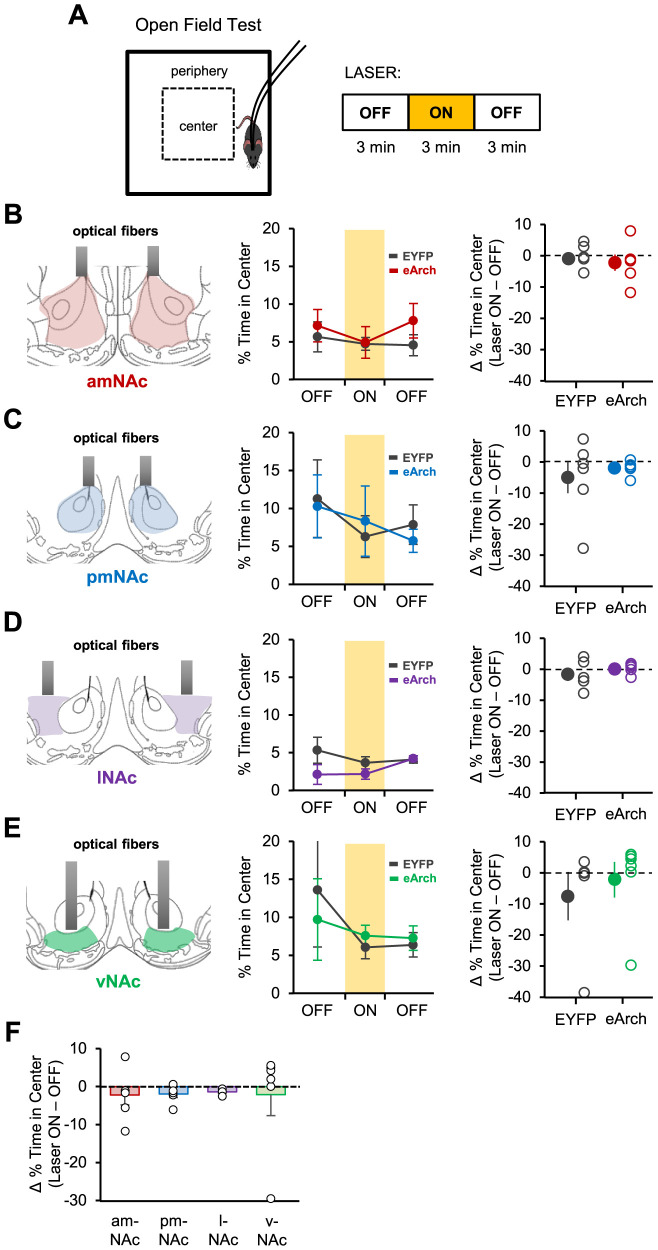
Inhibition of DA neuron terminals across NAc does not affect anxiety-like behavior in the open field test. ***A***, Schematic of the open field test and optogenetic inhibition protocol. ***B***, Left, Schematic of optogenetic inhibition in amNAc. Middle, Percent time eArch- (*n* = 6) and EYFP-expressing (*n* = 6) mice spent in the center of the open field during laser OFF and laser ON epochs of the test. Right, Difference between the percent of time mice spent in laser ON minus laser OFF epochs. The two groups exhibited comparable behavior (unpaired *t* test, *t*_(10)_ = 0.41, *p* = 0.68). ***C***, Left, Schematic of optogenetic inhibition in pmNAc. Middle, Percent time eArch- (*n* = 6) and EYFP-expressing (*n* = 6) mice spent in the center of the open field during laser OFF and laser ON epochs of the test. Right, Difference between the percent of time mice spent in laser ON minus laser OFF epochs. The two groups exhibited comparable behavior (unpaired *t* test, *t*_(10)_ = 0.59, *p* = 0.56). ***D***, Left, Schematic of optogenetic inhibition in lNAc. Middle, Percent time eArch- (*n* = 5) and EYFP-expressing (*n* = 5) mice spent in the center of the open field during laser OFF and laser ON epochs of the test. Right, Difference between the percent of time mice spent in laser ON minus laser OFF epochs. The two groups exhibited comparable behavior (unpaired *t* test, *t*_(8)_ = 0.76, *p* = 0.46). ***E***, Left, Schematic of optogenetic inhibition in vNAc. Middle, Percent time eArch- (*n* = 6) and EYFP-expressing (*n* = 5) mice spent in the center of the open field during laser OFF and laser ON epochs of the test. Right, Difference between the percent of time mice spent in laser ON minus laser OFF epochs. The two groups exhibited comparable behavior (unpaired *t* test, *t*_(9)_ = 0.58, *p* = 0.57). ***F***, Comparison of the difference between the percent time eArch-expressing mice spent in laser ON minus laser OFF side between the four groups. The four groups exhibited comparable levels of anxiety-like behavior during the laser OFF and ON epochs of the open field test (one-way ANOVA, *F*_(3,22)_ = 0.01, *p* = 0.99). Error bars represent mean ± SEM.

**Figure 7.**
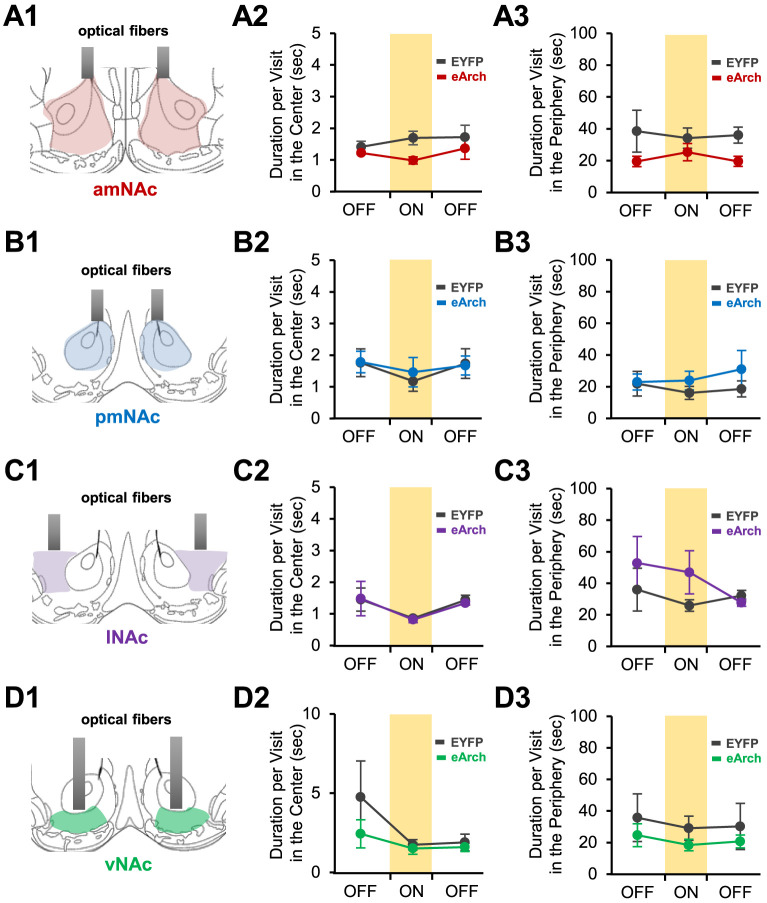
Analysis of average duration per visit in the center and periphery of the open field. ***A1*–*D1***, Schematic of optogenetic inhibition in amNAc, pmNAc, lNAc, and vNAc. ***A2*–*D2***, Average duration per visit in the center of the open field during laser OFF and ON epochs for EYFP end eArch groups. ***A3*–*D3***, Average duration per visit in the periphery of the open field during laser OFF and ON epochs for EYFP end eArch groups. Error bars represent mean ± SEM.

Open field test consisted of a session with three alternating epochs (OFF-ON-OFF epochs), during which laser was delivered in the ON epoch (Fig. 6*A*), as described above. In all four groups, neither eArch (amNAc: *n* = 6 males; pmNAc: *n* = 6 males; lNAc: *n* = 5 males; vNAc: *n* = 6, 4 males and 2 females) nor EYFP (amNAc: *n* = 6 males; pmNAc: *n* = 6 males; lNAc: *n* = 5 males; vNAc: *n* = 5, 4 males and 1 female) mice showed a significant change in their time spent in the center of the open field during the laser ON epoch (Fig. 6*B*–*E*). Two-way repeated-measures ANOVA comparing eArch and EYFP groups showed no effect of group for amNAc (*F*_(1,20)_ = 0.67, *p* = 0.43), pmNAc (*F*_(1,20)_ = 0.007, *p* = 0.93), lNAc (*F*_(1,16)_ = 2.92, *p* = 0.12), and vNAc (*F*_(1,18)_ = 0.02, *p* = 0.88). These results were further supported by comparing the change in center time between the first OFF and ON epoch in eArch- versus EYFP-expressing mice (Fig. 6*B*–*E*). Consistent with this, comparison of change in time spent in the center of the open field in eArch-expressing mice did not differ between the four groups (Fig. 6*F*). In addition, the average duration per visit to the center or the periphery of the open field (Fig. 6*A*) during the OFF and ON epochs were also comparable between the eArch- and EYFP-expressing mice for all four groups (Fig. 7*A*–*D*). Two-way repeated-measures ANOVA comparing eArch and EYFP groups showed no effect of group × trial interaction for amNAc (center: *F*_(2,16)_ = 0.62, *p* = 0.55; periphery: *F*_(2,16)_ = 0.29, *p* = 0.74), pmNAc (center: *F*_(2,20)_ = 0.12, *p* = 0.88; periphery: *F*_(2,20)_ = 0.47, *p* = 0.62), lNAc (center: *F*_(2,16)_ = 0.02, *p* = 0.97; periphery: *F*_(2,16)_ = 0.93, *p* = 0.41), and vNAc (center: *F*_(2,18)_ = 1.08, *p* = 0.35; periphery: *F*_(2,18)_ = 0.015, *p* = 0.98) groups. These results indicate that inhibition of DA projections across NAc subregions does not acutely affect anxiety-like behavior in the open field test.

### Inhibition of DA terminals across NAc does not acutely affect anxiety-like behavior in the EPM

**Figure 8.**
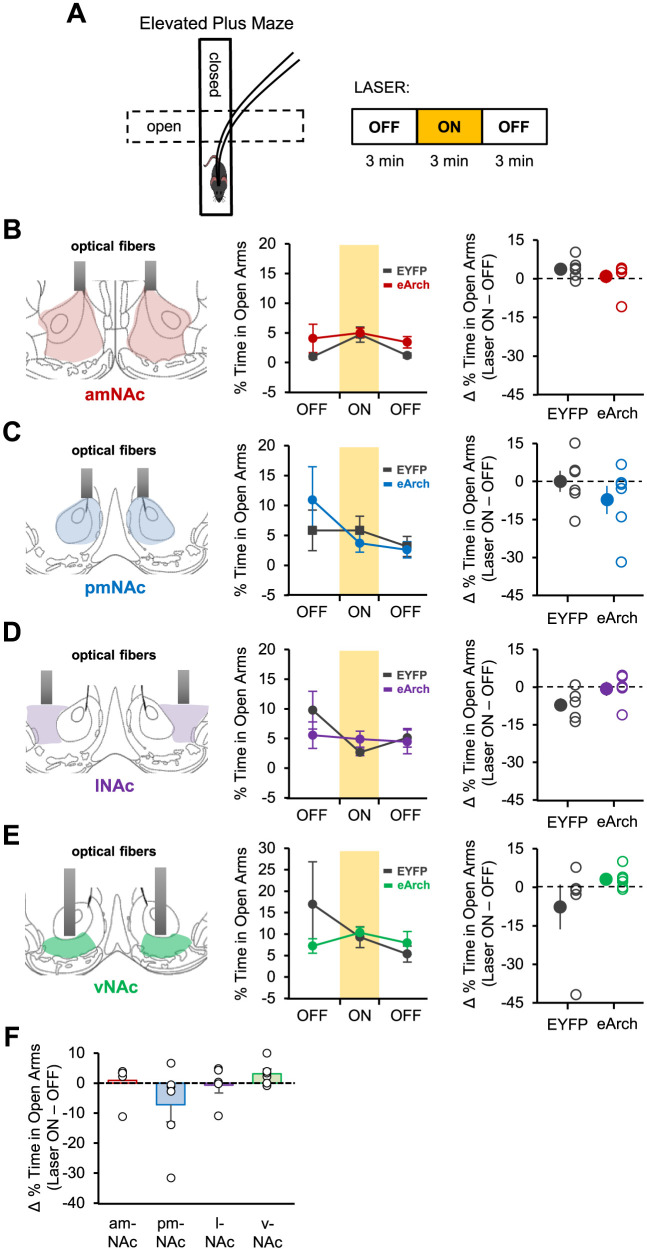
Inhibition of DA neuron terminals across NAc does not affect anxiety-like behavior in the EPM. ***A***, Schematic of the EPM test and optogenetic inhibition protocol. ***B***, Left, Schematic of optogenetic inhibition in amNAc. Middle, Percent time eArch- (*n* = 6) and EYFP-expressing (*n* = 6) mice spent in the open arms of the maze during laser OFF and laser ON epochs. Right, Difference between the percent of time mice spent in laser ON minus laser OFF epochs. The two groups exhibited comparable behavior (unpaired *t* test, *t*_(10)_ = 0.96, *p* = 0.35). ***C***, Left, schematic of optogenetic inhibition in pmNAc. Middle, Percent time eArch- (*n* = 6) and EYFP-expressing (*n* = 6) mice spent in the open arms of the maze during laser OFF and laser ON epochs. Right, Difference between the percent of time mice spent in laser ON minus laser OFF epochs. The two groups exhibited comparable behavior (unpaired *t* test, *t*_(10)_ = 1.03, *p* = 0.32). ***D***, Left, Schematic of optogenetic inhibition in lNAc. Middle, Percent time eArch- (*n* = 5) and EYFP-expressing (*n* = 5) mice spent in the open arms of the maze during laser OFF and laser ON epochs. Right, Difference between the percent of time mice spent in laser ON minus laser OFF epochs. The two groups exhibited comparable behavior (unpaired *t* test, *t*_(8)_ = 1.66, *p* = 0.13). ***E***, Left, Schematic of optogenetic inhibition in vNAc. Middle, Percent time eArch- (*n* = 6) and EYFP-expressing (*n* = 5) mice spent in the open arms of the maze during laser OFF and laser ON epochs. Right, Difference between the percent of time mice spent in laser ON minus laser OFF epochs. The two groups exhibited comparable behavior (unpaired *t* test, *t*_(9)_ = 1.34, *p* = 0.21). ***F***, Comparison of the difference between the percent time eArch-expressing mice spent in laser ON minus laser OFF side between the four groups. The four groups exhibited comparable levels of anxiety-like behavior during the laser OFF and ON epochs of the EPM test (one-way ANOVA, *F*_(3,22)_ = 1.67, *p* = 0.20). Error bars represent mean ± SEM.

To determine whether the lack of an effect on anxiety-like behavior in the open field test was consistent across different behavioral paradigms, we next tested animals in the EPM, another widely used test for assessing anxiety in rodents. Similar to the open field, EPM test also consisted of a session with three alternating epochs (OFF-ON-OFF epochs), during which laser was delivered in the ON epoch (Fig. 8*A*).

**Figure 9.**
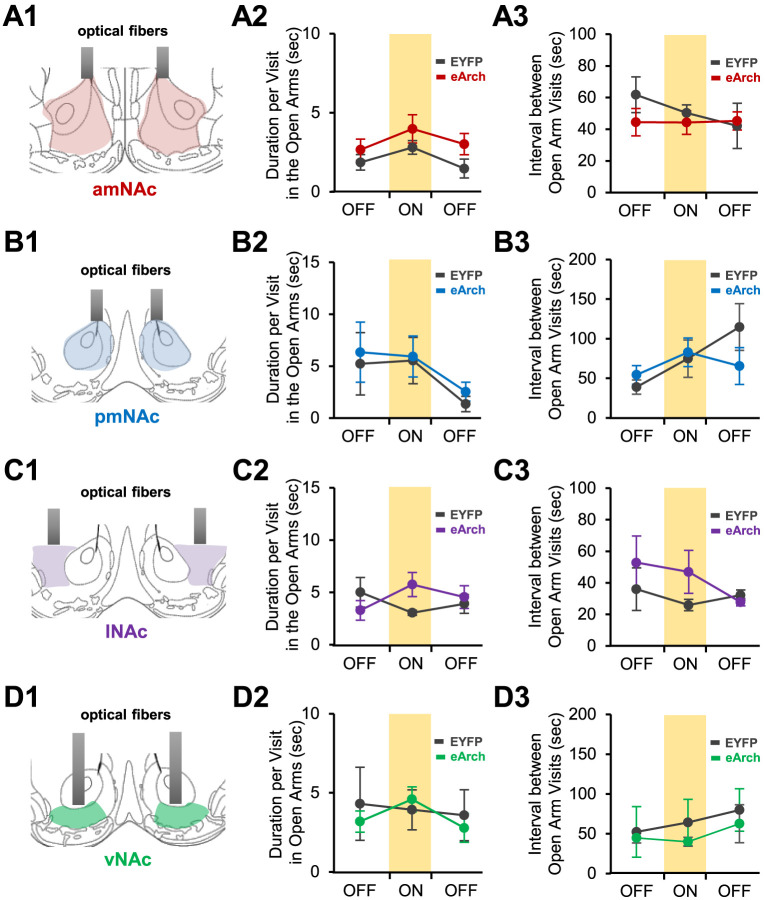
Analysis of average duration per visit in the open arms of the EPM. ***A1*–*D1***, Schematic of optogenetic inhibition in amNAc, pmNAc, lNAc, and vNAc. ***A2*–*D*2**, Average duration per visit in the open arms of the EPM during laser OFF and ON epochs for EYFP and eArch groups. ***A3*–*D3***, Average interval between open arm visits in the EPM during laser OFF and ON epochs for EYFP end eArch groups. Error bars represent mean ± SEM.

In all four groups, eArch (amNAc: *n* = 6 males; pmNAc: *n* = 6 males; lNAc: *n* = 5 males; vNAc: *n* = 6, 4 males and 2 females) and EYFP (amNAc: *n* = 6 males; pmNAc: *n* = 6 males; lNAc: *n* = 5 males; vNAc: *n* = 5, 4 males and 1 female) mice did not show a significant change in time spent in the open arms of the EPM during the laser ON epoch (Fig. 8*B*–*E*). Two-way repeated-measures ANOVA comparing eArch and EYFP groups showed no effect of group for amNAc (*F*_(1,20)_ = 3.51, *p* = 0.09), pmNAc (*F*_(1,20)_ = 0.09, *p* = 0.76), lNAc (*F*_(1,16)_ = 0.30, *p* = 0.59), and vNAc (*F*_(1,18)_ = 0.26, *p* = 0.61). Comparison of the change in open arm time between the first OFF and ON epoch in eArch- versus EYFP-expressing mice further confirmed these findings (Fig. 8*B*–*E*). Consistent with this, comparison of change in time spent in the open arms of the maze in eArch-expressing mice did not differ between the four groups (Fig. 8*F*). Moreover, the average duration per visit to the open arms and the average interval between open arm visits during the OFF versus ON epochs were also comparable between the eArch- and EYFP-expressing mice for all four groups (Fig. 9*A*–*D*). Two-way repeated-measures ANOVA comparing eArch and EYFP groups showed no effect of group x trial interaction for amNAc (open arm: *F*_(2,16)_ = 0.21, *p* = 0.1; interval: *F*_(2,16)_ = 0.54, *p* = 0.68), pmNAc (open arm: *F*_(2,20)_ = 0.02, *p* = 0.97; interval: *F*_(2,20)_ = 1.86, *p* = 0.18), lNAc (open arm: *F*_(2,16)_ = 2.09, *p* = 0.15; interval: *F*_(2,16)_ = 0.29, *p* = 0.75), and vNAc (open arm: *F*_(2,18)_ = 0.33, *p* = 0.73; interval: *F*_(2,18)_ = 0.30, *p* = 0.74) groups. These results indicate that inhibition of DA projections across NAc subregions does not acutely affect anxiety-like behavior in the EPM. Taken together, these results suggest that dopaminergic input across different NAc subregions likely does not play a significant role in modulating anxiety-like behaviors as evaluated across a range of behavioral tests.

## Discussion

DA projections to specific subregions of the NAc have been shown to be activated by aversive events (de Jong et al., 2019; Yuan et al., 2019; Kutlu et al., 2023; Salinas-Hernández et al., 2023). However, it remains unclear whether DA input to these NAc subregions is intrinsically aversive and whether such signaling contributes to modulation of anxiety-like behaviors. Here, using predominantly male mice, we demonstrated that inhibition of DA projections across all NAc subregions produced robust place avoidance, indicating that inhibiting DA input to each subregion is similarly aversive and that these DA projections are intrinsically reinforcing. In contrast, DA terminal inhibition in NAc resulted in subregion-specific effects on locomotor activity, with the largest influence observed in lNAc. Finally, inhibition of DA projections across NAc subregions did not have an acute effect on anxiety-like behavior, suggesting that DA input to NAc does not play a major role in the acute modulation of anxiety.

The mesolimbic DA system has traditionally been associated with reward processing (Wise, 2002; Berridge and Kringelbach, 2015; Schultz, 2016; Watabe-Uchida et al., 2017). Consistent with this, VTA DA neurons and their projections to NAc have been shown to be activated by rewards and exhibit RPE signals (Parker et al., 2016; Schultz, 2016; Menegas et al., 2017; Watabe-Uchida, et al., 2017; de Jong et al., 2019; Tsutsui-Kimura et al., 2020; Salinas-Hernández et al., 2023). Furthermore, activation of VTA DA neurons as well as their projections to NAc have consistently been found to be intrinsically reinforcing (Tsai et al., 2009; McDevitt et al., 2014; Cardozo Pinto et al., 2025). However, recent studies have revealed that DA neurons projecting to NAc are not functionally uniform but rather display heterogeneity depending on the NAc subregion they innervate. In particular, DA projections to the ventral subareas of NAc have been shown to be activated by aversive stimuli and threat-predicting cues, suggesting that these DA terminals might encode aversion per se (de Jong et al., 2019; Yuan et al., 2019; Salinas-Hernández et al., 2023). However, contrary to this notion, we found that inhibition of DA terminals in vNAc caused robust place avoidance in a real-time place preference task. In line with our results, these DA terminals have been shown to be activated by both aversive and rewarding outcomes (de Jong et al., 2019; Yuan et al., 2019; Salinas-Hernández et al., 2023), suggesting that they unlikely encode aversive value. Overall, our results indicate that DA projections to vNAc are not intrinsically aversive but instead promote mainly positively rather than negatively reinforced behaviors.

Previous studies have revealed a functional segregation along the anteroposterior axis of the medial NAc (mNAc), such that the anterior (amNAc) and posterior (pmNAc) subregions are differentially involved in processing appetitive and aversive outcomes, respectively (Reynolds and Berridge, 2001, 2003; Berridge and Kringelbach, 2013; Castro and Berridge, 2014; Castro et al., 2016; Reed et al., 2018). Consistent with this anatomical and functional gradient, a recent study demonstrated that DA projections to mNAc also display an anteroposterior gradient in their responses to aversive and safe outcomes (Salinas-Hernández et al., 2023). Despite this reported segregation, we found that inhibition of DA terminals in both amNAc and pmNAc produced place avoidance in a real-time place preference task, indicating that DA inputs to both subregions are intrinsically reinforcing. Notably, the comparable magnitude of place avoidance between the two groups suggests that suppression of DA input to the amNAc and pmNAc are equally aversive and that both DA projections are reinforcing. In support of our findings, previous work has shown that DA terminals in the amNAc and pmNAc are activated by rewards to a similar extent (Salinas-Hernández et al., 2023).

Our study provides a comprehensive characterization of the role of DA projections across NAc subregions in promoting reward-like behavior. We found that inhibition of DA terminals across all subregions of NAc induced robust place avoidance, indicating that DA input throughout NAc is generally intrinsically reinforcing, whereas its suppression elicits aversion-related behavior. Our results are in line with previous studies demonstrating the causal role of VTA DA neurons and their projections to NAc in place preference paradigms. Specifically, activation of VTA DA neurons and their terminals in NAc reliably induces place preference (Tsai et al., 2009; McDevitt et al., 2014; Cardozo Pinto et al., 2025), whereas their inhibition produces place aversion (Tan et al., 2012; Danjo et al., 2014; Cai et al., 2020). Furthermore, dopamine terminals in the ventral striatum have been consistently shown to exhibit phasic activation in response to rewards, notably across all recorded NAc subregions (Parker et al., 2016; Menegas et al., 2017; de Jong et al., 2019; Yuan et al., 2019; Tsutsui-Kimura et al., 2020; Salinas-Hernández et al., 2023). Consistent with this, a recent study found that the majority of VTA DA neurons exhibit uniform activation to rewards (Engelhard et al., 2019). Taken together, these findings suggest that DA neurons projecting across different subregions of NAc exhibit a largely homogeneous activation to rewards, and their activity is intrinsically reinforcing.

DA neurons, particularly those projecting to the dorsal striatum, play a central role in motor control (Surmeier et al., 2009). However, despite the well-established involvement of DA neurons in modulation of movement, the contribution of DA projections to the NAc in locomotor behavior remains incompletely understood. Previous studies using optogenetic manipulation of VTA DA neurons or their projections to NAc have reported conflicted findings. Specifically, while optogenetic activation of VTA DA neurons have been reported to either have no effect on locomotor behavior (Tsai et al., 2009; Gunaydin et al., 2014) or increase locomotion (Kim et al., 2012), activation of DA terminals in the posteromedial shell of NAc has been shown to increase locomotion (Cardozo Pinto et al., 2025). Interestingly, although broad optogenetic inhibition of VTA DA neurons has been reported to have no effect on locomotor activity (Tan et al., 2012; Gunaydin et al., 2014), selective inhibition of DA neurons in either the medial or lateral VTA decreased velocity (Cai et al., 2020). These findings suggest that the specific DA neuron subpopulations targeted within the VTA can differentially affect the behavioral outcome. At the level of DA terminals, inhibition of DA projections to the posteromedial shell of NAc was previously reported to have no effect on locomotor activity (Cardozo Pinto et al., 2025). In our study, we found that inhibition of DA terminals increased velocity in the amNAc, pmNAc, and lNAc, while having no effect in the vNAc. Notably, these effects were observed consistently across multiple behavioral tests, including the place preference and open field. One potential explanation for these inconsistencies in the literature is the considerable heterogeneity of VTA DA neurons. Differences in the specific DA neuron populations or their projection-defined circuits targeted across studies may account for some of the variability in reported findings. In addition, locomotor activity can be influenced not only by motor processes but also by motivational, exploratory, and affective states. For example, the increased locomotor activity observed in our study may represent enhanced exploratory behavior or an active behavioral response to the aversive state induced by inhibition of DA signaling. Differences in the extent to which specific DA circuits influence these behavioral states could further contribute to the variability in locomotor activity reported across studies. Overall, our findings indicate that DA projections to distinct NAc subregions differentially modulate movement, highlighting the functional heterogeneity within mesolimbic DA circuits in controlling locomotor behavior.

VTA DA neurons and their projections to NAc have been implicated in regulation of affective states such as stress and depression (Berton et al., 2006; Tye et al., 2013; Heshmati and Russo, 2015; Willmore et al., 2022; Nestler and Russo, 2024). Given that DA neurons projecting to specific NAc subregions are activated by aversive stimuli and threats, determining whether DA input to the NAc also modulates anxiety-like behavior, particularly in a subregion-specific manner, is a critical question for understanding the neural circuits underlying negative affective states. However, activation of VTA DA neurons has produced inconsistent results, with one study reporting no effect (Tsai et al., 2009) and another demonstrating increased anxiety-like behavior that is mainly mediated by DA projections to the medial prefrontal cortex (mPFC; Gunaydin et al., 2014). Whether DA projections to NAc contribute to anxiety-related behavior remained elusive. In the present study, we performed two well-established anxiety tests to examine the role of DA projections to NAc subregions in anxiety-like behavior. The open field test was used to evaluate exploratory behavior in an open, novel environment, whereas the EPM test measured anxiety-like behavior based on aversion to open, elevated spaces. Our results indicate that optogenetic inhibition of DA terminals across NAc subregions did not have an acute effect on anxiety-like behavior in the tests used in this study. However, because the present study examined only the acute effects of optogenetic inhibition and did not assess potential long-term effects on synaptic plasticity and learning-related processes, our findings do not rule out a role for the mesolimbic DA system in the long-term modulation of anxiety-related behaviors or in other behavioral paradigms. Nevertheless, our findings are consistent with previous studies (Tsai et al., 2009; Gunaydin et al., 2014) and suggest that the regulation of anxiety-like behavior is likely mediated primarily by DA projections to regions outside the NAc, such as the mPFC.

Several limitations of the present study should be considered. First, although eArch is widely used for optogenetic terminal inhibition, prolonged illumination of eArch-expressing axon terminals has been reported to increase presynaptic calcium levels and spontaneous neurotransmitter release (Mahn et al., 2016). While laser stimulation in the real-time place preference task occurred in brief bouts (average 10–20 s) and the stimulation periods used in our open field and EPM experiments were shorter than those shown to induce such effects, we cannot exclude the possibility that some behavioral effects were influenced by mechanisms beyond simple inhibition. Furthermore, sustained manipulations may potentially produce effects that persist beyond the stimulation period, including increased unsynchronized neurotransmitter release and rebound changes in neuronal activity (Lafferty and Britt, 2020). These persistent effects may account for the sustained increase in velocity observed in our study. Nevertheless, as place avoidance and changes in locomotor activity were observed during short-duration laser stimulations in the real-time place preference test, our major findings are likely not driven by secondary effects associated with prolonged terminal inhibition. Second, although optical fibers were positioned directly above the targeted NAc subregion and laser power was maintained within a range commonly used for terminal inhibition experiments, light diffusion beyond the target subregion cannot be ruled out. Furthermore, DAergic axons passing by could also have been affected. Nevertheless, the distinct behavioral effects observed following inhibition of DA terminals in different NAc subregions, particularly the differential effects on locomotor activity, suggest that the manipulations produced subregion-specific effects. Finally, because DAT expression is absent or expressed at low levels in some midbrain DA neurons (Lammel et al., 2008; Anderegg et al., 2015), the DAT-Cre mouse line used in this study likely targeted the majority, but not all, of DA neurons projecting to the NAc. Consequently, our manipulations likely affected most, but not all, DA projections to the NAc subregions. These technical limitations should be considered when interpreting the circuit- and subregion-specific effects reported here.

Overall, our study reveals that dopaminergic projections across different NAc subregions, despite their heterogeneous activation in response to aversive and safety-related stimuli, are uniformly reinforcing and their inhibition is intrinsically aversive and induces avoidance. In contrast, the effects on locomotor activity were subregion specific, and DA input to the NAc did not acutely affect anxiety-like behavior. These results suggest that mesolimbic DA signaling acts primarily to promote appetitive processes. More broadly, our results indicate the functional specialization of projection-defined DA circuits and highlight the importance of both projection-specific and subregion-defined approaches for elucidating the diverse functional contributions of the mesolimbic DA system.

## Synthesis

Reviewing Editor: Maithe Arruda-Carvalho, University of Toronto Scarborough Decisions are customarily a result of the Reviewing Editor and the peer reviewers coming together and discussing their recommendations until a consensus is reached. When revisions are invited, a fact-based synthesis statement explaining their decision and outlining what is needed to prepare a revision will be listed below. The following reviewer(s) agreed to reveal their identity: Mitsuko Watabe-Uchida, Jonathan Britt. Note: If this manuscript was transferred from JNeurosci and a decision was made to accept the manuscript without peer review, a synthesis may not be available.

This study by Salinas-Hernandez et al. examined the effects of optogenetic inactivation of dopamine axons in 4 nucleus accumbens (NAc) subregions (anteromedial, posteromedial, lateral and ventral) on mouse behaviors in 3 behavioral paradigms: real-time place preference, open field and elevated plus maze.

The experiments are carefully performed, results are well presented and the text is easy to follow with proper citations. The results are important and timely, especially given how much conflicting data there is in the literature concerning the behavioral impact of dopamine signaling across nucleus accumbens subregions. The experiments are somewhat underpowered (n=6/group), but the overall message is clear, and the data will certainly be of interest to anyone studying the function of the nucleus accumbens. Below are the main points raised by the reviewers to be addressed by the authors:

Major suggestions:

Experimental:

- Validation of terminal inhibition. Authors intended to inhibit dopamine release at NAc subregions by locally stimulating dopamine axons expressing eArch3.0. However, they never verified the optogenetic effects on dopamine release in each subregion. Even though the fiber placement is OK, it is not clear whether their optical stimulation has induced local decrease of dopamine release as they wanted because it might stimulate passing fibers to other subregions. Second, presynaptic stimulation may cause complicated side effects such as increase of unsynchronized neurotransmitter release in vitro and in vivo (see Lafferty and Britt, 2020 and others). Third, sustained stimulation like this study may cause rebound activity or sustained effects beyond the stimulation period on those neurons and circuits. To directly address these issues, it is necessary to provide data validating the impact of the optic manipulation (e.g with fiber photometry) in at least one subregion. Given that the optogenetic effects on locomotion lasted longer than the duration of optogenetic inhibition, the recordings should also examine potential sustained effects of dopamine axon stimulation for 3 min on dopamine release after the stimulation period. The authors should integrate the potential caveats raised above in their discussion.

Data analysis/Presentation:

- It is interesting that optogenetic inhibition of dopamine axons in all NAc subregions showed similar effects on place avoidance. To distinguish the causes of avoidance, it is useful if authors quantify duration per visit, and the interval between visits to the stimulation side.

- Mere increase of locomotion may partly explain place "avoidance" because increased random locomotion in the stimulation site may push animals away from there. While duration per visit, just mentioned above, addresses this, it is also useful to quantify distance travelled in each side.

- Similarly, it is useful to quantify duration per visit, and the interval between visits to the center of the arena and to the open arm.

- In the example images of Fig 1, the fiber tip should be visible (instead of covered by with a black bar).

- The authors use male and female mice, but sex differences were not assessed. All data should be analyzed for sex. As they may be underpowered to detect sex differences, this should be acknowledged and discussed in the manuscript. The exact breakdown of male and female mice should be provided for each experiment, and the authors should consider visually distinguishing individual datapoints according to sex.

Discussion/Text changes:

- Because authors only examined acute effects of dopamine, some conclusions are too strong. Dopamine has sustained and learning effects in addition to acute effects. Especially, "mesolimbic DA pathway does not play a significant role in modulating anxiety-related behaviors" at line 21, page 16 in Discussion and similar statements in other places should be rephrased.

- The Discussion section should include a paragraph addressing technical considerations, such as the use of Arch for axon terminal inhibition (see Mahn et al., 2016), whether the light power (5-10 mW) was sufficiently low to ensure subregion-specific inhibition, and the use of DAT-Cre mice (given that DAT is expressed in most but not all dopamine neurons (see Anderegg et al., 2015)).

- The data presented show that dopamine axon photo-inhibition throughout the nucleus accumbens reliably elicits escape/avoidance behavior, but this does not necessarily mean that "DA input to each subregion is similarly rewarding." That statement requires tests of subregion-specific dopamine axon stimulation. Moreover, in many places in the manuscript, it may be more appropriate to use the word 'reinforcing' rather than 'rewarding', given the longstanding debate about relationship between dopamine signaling and hedonic pleasure.

- The authors should be more circumspect in some of their conclusions, given the conflicting data in this field. The presented data show that acute photo-inhibition does not reliably alter anxiety-related behaviours in the moment or over the subsequent few minutes, but these data do not completely rule out a role for these pathways in regulating anxiety in other assays or over longer periods of time via learning. Also, the interpretation that ventral nucleus accumbens dopamine encodes motivational salience is difficult to reconcile with the observed place avoidance, which instead suggests that reduced dopamine signaling is perceived as aversive.

Minor suggestions:

- In the real-time place preference task, is the animals' avoidance of the light-paired side similar across the two 10-minute epochs (before and after the reversal). If there is a significant difference between those time periods, it would be nice to see the data separated in that manner.

- It is surprising that dopamine axon inhibition increased locomotor behavior. The authors should speculate about why this behavioral measure is so variable across similar studies.

- In Methods, please cite original papers for the virus constructs.
